# Improving CORM technology for the treatment of delayed hemolytic transfusion reaction

**DOI:** 10.1002/hem3.140

**Published:** 2024-08-05

**Authors:** Michela Asperti, Francesca Vinchi

**Affiliations:** ^1^ Iron Research Laboratory, Lindsley Kimball Research Institute New York Blood Center New York New York USA; ^2^ Department of Molecular and Translational Medicine University of Brescia Brescia Italy; ^3^ Department of Pathology and Laboratory Medicine Weill Cornell Medicine New York New York USA

Delayed hemolytic transfusion reaction (DHTR) is a severe and potentially fatal complication triggered by red blood cells (RBC) transfusions^1^ in patients with sickle cell disease (SCD). Transfusions remain a major therapeutic intervention in the clinical management of anemia as well as both acute and chronic disease‐related complications in SCD.^1^, ^2^, ^3^ Typically, DHTR occurs days to weeks after a RBC transfusion due to the sudden destruction of both transfused and patients' RBCs, with a consequent drastic drop in hemoglobin (Hb), seriously threatening the life of SCD patients.^4^, ^5^ During DHTR with hyperhemolysis, the release of free Hb and heme has deleterious impact on the vasculature, causing vasculo‐toxicity and leading to vasculopathy due to intravascular oxidative stress, endothelial damage, increased expression of proadhesive, proinflammatory and chemotactic factors and reduced nitric oxide (NO) bioavailability. Upon RBC exposure, one or more alloantibodies are produced in SCD patients, which contribute to DHTR. In one‐third of RBC transfused patients, complement activation—rather than alloantibodies production—plays a role in DHTR, both through the canonic pathway, whereby complement fixed antibody binds to RBCs, and the alternative pathway, whereby free heme‐induced TLR4 signaling on endothelial cells activates the complement system.^1^ Patients experience symptoms such as fever, pain, fatigue, mild jaundice or dark urine and a drastic Hb drop. The current treatment options for DHTRs are based on supportive care, erythropoiesis optimization, immunomodulatory treatments, including complement inhibition, steroids, intravenous immunoglobulin, and/or B cell depletion, and future transfusion avoidance, even if the latest may be not always feasible in some clinical conditions related to cardiac or respiratory failure.^1^, ^3^

Among the therapeutic strategies proposed to overcome DHTR, carbon monoxide administration in the form of inhalation or carbon‐monoxide‐releasing molecules (CO‐RMs) has shown promising results in preclinical studies.^6^ A plethora of CORMs has been generated, structurally designed with a central transition metal such as iron, manganese, or cobalt, surrounded by CO as a ligand.^6^ CO is a stable molecule that is continuously produced after the catabolism of heme by heme‐oxygenases (HO), a family of enzymes with established anti‐inflammatory and cytoprotective functions. Mechanistically, CO decreases the expression of proinflammatory and increases the expression of anti‐inflammatory cytokines by activating the MKK3/p38β MAPK pathway and inducing PPARγ. In addition, it reduces TLR4 activation by inhibiting TLR4 trafficking, and its interaction with caveolin‐1 at the plasma membrane. CO also serves as a bioactive signaling molecule acting as intracellular mediator in a variety of physiological functions, including vasodilation and cardiac protection through the activation of the soluble guanylyl cyclase, regulation of the nervous system through the activation of potassium channels, and control of neurotransmitters, as well as gastrointestinal and respiratory tracts.^7^

In vitro as well as preclinical studies provide evidence of significant anti‐inflammatory, antioxidant, and antiapoptotic effects as well as vasodilation and antiadhesion action of CO on the vasculature, resulting in the preservation of vascular flow, with major implications for SCD.^8^ In this context, Nguyen Kim‐Anh and coauthors recently reported the beneficial effect of CORM‐401 on endothelial activation, tissue damage, and inflammation caused by acute hyperhemolysis in SCD.^9^ CORM‐401 has the capability to carry and deliver controlled amounts of CO to biological systems leading to the activation of endothelial calcium signaling and increased NO bioavailability, with consequent therapeutic benefit.^6^, ^10^, ^11^, ^12^

The novelty of this study lies in the development of in vitro and in vivo models that reflect endothelial damage and organ dysfunction occurring during the early phase of hyper‐hemolysis in SCD. The authors reproduced vascular activation and dysfunction in the early DHTR phase taking advantage of a new in vitro fluidic model whereby umbilical vein endothelial cells (HUVEC) were exposed to hemolysates containing RBC membrane‐derived particles with negligible levels of free oxidized hemoglobin or heme.^9^ The pre‐exposure of HUVEC cells to CORM‐401 significantly increased the content of COHb and prevented hemolysates‐induced upregulation of proinflammatory cytokines such as IL6, IL1, and IL8, and adhesive molecules, including Vascular and Intercellular Cell Adhesion Molecules‐1 (VCAM‐1, ICAM‐1). Moreover, due to an overall reduction of oxidative stress, it inhibited the expression of the acute phase redox‐sensitive transcription factor nuclear factor erythroid‐2‐related factor 2, Nrf2.^9^

To corroborate in vitro findings, the authors evaluated the beneficial effects of the CO‐released by CORM‐401 in a humanized SCD mouse model exposed to hemolysate. CORM‐401 effectively released CO in several tissues, including liver, kidney, lung, cecum, and colon, with no sign of toxicity. Pretreatment of SCD mice with CORM‐401^9^ prevented hemolysate‐induced acute damage in target organs commonly affected during DHTR, namely lung, liver, and kidney. In particular, CORM‐401 efficiently resolved inflammation by modulating NF‐κB and Nrf2 signaling pathways, and the expression of downstream target genes, including proinflammatory and adhesive molecules (VCAM‐1, ICAM‐1, endothelin‐1, thromboxane) and antioxidants enzymes (HO‐1, Gpx1).^9^ In detail, CORM‐401 administration provided a protective effect against lung injury, by preventing alveolar dilation and septum wall thickening due to hemolysate exposure. Overall, CORM‐401 decreased inflammatory cell infiltrates, thrombi formation, and macrophage iron accumulation in the lung. CORM‐401 decreased the active form of Nrf2 and NF‐kB, reduced HO‐1 and thromboxane synthase (TBXS) expression, and decreased VCAM‐1, ICAM‐1, and ET‐1, important for the endothelial cell activation. Similarly, CORM‐401 treatment increased CO content in the liver with consequent reduction of inflammation, thrombi formation, and iron accumulation in hepatic macrophages. A significant reduction of hepatic Nrf2 and NF‐ kB was also observed in the liver. Kidney is another organ affected by the hemolysate‐induced acute damage and CORM‐401 treatment led to a reduction in glomerular inflammatory cell infiltration, and a mild decrement in tubular iron accumulation. Nrf2 activation, ICAM‐1 expression as well as the induction of the antioxidant enzymes HO‐1 and NAD(P)H dehydrogenase (quinone)‐1 were reduced by CORM‐401 therapy. CORM‐401 was also effective in suppressing the induction of P‐Selectin and VCAM‐1 in SCD exposed to hemolysate, protecting against hemolysate‐induced vascular dysfunction.^9^ In summary in a DHTR model in SCD mice, CORM‐401 treatment showed therapeutic benefits, limiting organ damage in commonly affected tissues, and ameliorating endothelial dysfunction and inflammatory vasculopathy Figure 1.

**Figure 1:**
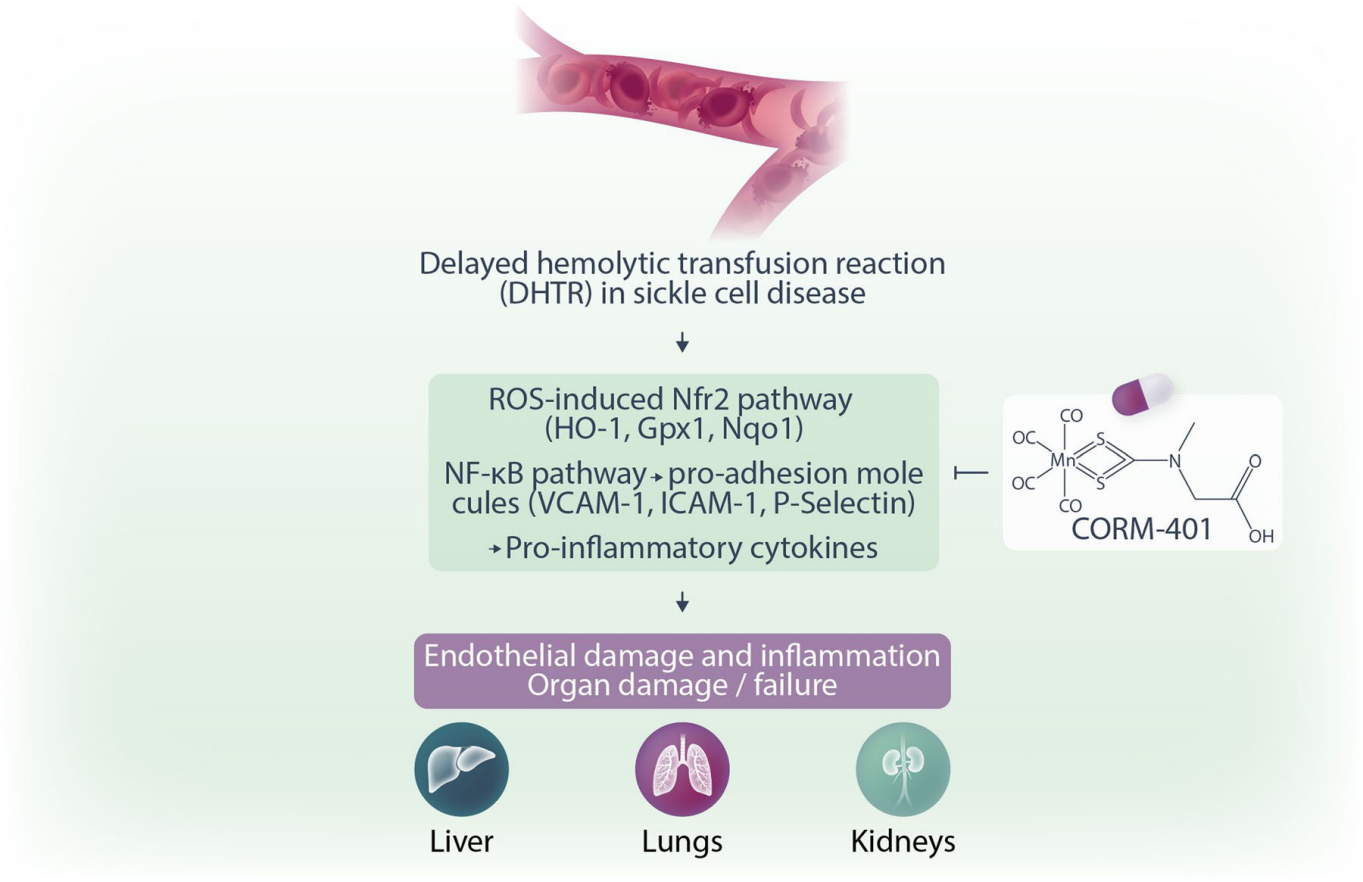
CORM‐401 protects against hemolysis‐driven tissue injury and inflammation upon delayed hemolytic transfusion reaction (DHTR). During DHTR, erythrocyte microparticles induce hemolysis‐driven injury affecting the vascular endothelium and multiple organs, including liver, kidney, and lung. CORM‐401 blocks the detrimental effects of acute hemolysis by limiting the activation of the NF‐κB and Nrf2 pathways, reducing inflammation, and preventing vasculo‐toxicity and tissue injury, with potential therapeutic effects in sickle cell disease patients experiencing or prone to DHTR.

The advent of CORMs significantly improved the route of CO administration, allowing the delivery of controlled and not‐toxic amount of CO to tissues.^6^ This significantly augmented the potential to use CO‐related therapies in SCD for the amelioration of inflammatory vasculopathy and the prevention of vaso‐occlusive crises.^8^ This study provides a proof of concept for the applications of CORMs in severe sickle complications such as DHTR. Limiting early DHTR consequences is key to the prevention of end‐organ damage, and CORM‐401 exhibited extensive therapeutic benefit in multiple organs. The additional advantages of the specific Mn‐containing CORM‐401 consist in the easy chemical synthesis, increased water solubility, and stability of the compound, combined to a safe CO delivery with no significant toxic or adverse events. While most CORMs tested in SCD mainly exhibited beneficial effects on hemolysis‐driven endothelial dysfunction, this study shows for the first time a broader protective effect of CORMs against hemolysis‐induced tissue injury.^9^ Overall, the reduction of the deleterious effects of acute hyperhemolysis—rather than the toxicities associated with chronic hemolysis—suggests that CORMs can be valuable therapeutic approaches for the treatment and eventually prevention of acute high‐risk complications such as DHTR in SCD patients. Whether combination treatment with this novel CORM and other agents such as heme scavengers or antisickling compounds provides further advantage for both chronic hemolysis and hyperhemolytic events remains to be explored.

## AUTHOR CONTRIBUTIONS

Michela Asperti and Francesca Vinchi wrote the HemaTopic and drafted the figure, which was professionally drawn by Somersault18:24 BV.

## CONFLICT OF INTEREST STATEMENT

Dr. Vinchi is a member of the advisory board of Silence Therapeutics and a consultant for RallyBio and Pharmacosmos. None of these relationships is relevant to the current publication.

## FUNDING

This research received no funding.

## Data Availability

Data sharing not applicable to this article as no data sets were generated or analysed during the current study.
